# Lectin Activity of the TcdA and TcdB Toxins of Clostridium difficile

**DOI:** 10.1128/IAI.00676-18

**Published:** 2019-02-21

**Authors:** Lauren E. Hartley-Tassell, Milena M. Awad, Kate L. Seib, Maria Scarselli, Silvana Savino, Joe Tiralongo, Dena Lyras, Christopher J. Day, Michael P. Jennings

**Affiliations:** a Institute for Glycomics, Griffith University, Gold Coast, Australia; b Infection and Immunity Program, Biomedicine Discovery Institute and Department of Microbiology, Monash University, Clayton, Australia; c GSK Vaccines, Siena, Italy; University of Michigan—Ann Arbor

**Keywords:** *Clostridium difficile*, host cell interactions, pathogenesis, toxin-receptor interaction

## Abstract

Clostridium difficile is a major cause of hospital-acquired antibiotic-associated diarrhea. C. difficile produces two cytotoxins, TcdA and TcdB; both toxins are multidomain proteins that lead to cytotoxicity through the modification and inactivation of small GTPases of the Rho/Rac family. Previous studies have indicated that host glycans are targets for TcdA and TcdB, with interactions thought to be with both α- and β-linked galactose. In the current study, screening of glycan arrays with different domains of TcdA and TcdB revealed that the binding regions of both toxins interact with a wider range of host glycoconjugates than just terminal α- and β-linked galactose, including blood groups, Lewis antigens, *N*-acetylglucosamine, mannose, and glycosaminoglycans. The interactions of TcdA and TcdB with ABO blood group and Lewis antigens were assessed by surface plasmon resonance (SPR). The blood group A antigen was the highest-affinity ligand for both toxins. Free glycans alone or in combination were unable to abolish Vero cell cytotoxicity by TcdB. SPR competition assays indicate that there is more than one glycan binding site on TcdB. Host glycoconjugates are common targets of bacterial toxins, but typically this binding is to a specific structure or related structures. The binding of TcdA and TcdB is to a wide range of host glycans providing a wide range of target cells and tissues *in vivo*.

## Abstract (precis)

Clostridium difficile is a major cause of hospital-acquired antibiotic-associated diarrhea. C. difficile produces two cytotoxins, TcdA and TcdB; both toxins are multidomain proteins that lead to cytotoxicity through the modification and inactivation of small GTPases of the Rho/Rac family.

## INTRODUCTION

Clostridium difficile infections (CDIs) place a large disease and financial burden on health care systems since C. difficile is the major cause of hospital-acquired diarrhea worldwide. CDI incidence and severity have increased since the early 2000s (1, 2), prompting research efforts to identify therapeutics that may act as alternatives to antibiotics, which remain the current best standard-of-care treatment. C. difficile produces two major cytotoxins, TcdA and TcdB (TcdA/B), with TcdB thought to be largely responsible for the gut damage that occurs during CDI (3, 4). Both toxins are monoglucosyltransferases that form part of the family of large clostridial toxins, or LCTs. These toxins modify and inactivate small GTPases of the Rho/Rac family, leading to colonic inflammation, tissue damage, and ultimately cell death (5, 6).

TcdA and TcdB are multidomain proteins that consist of at least four functionally distinct regions (A, C, D, and B) (7–9). These include the glucosyltransferase domain (GTD) (domain A) that is responsible for inactivating small Rho-dependent GTPases, the cysteine protease domain (CPD) (domain C) required for proteolytic cleavage of the toxins, the delivery domain (DD) (domain D) that enables the translocation of the N terminus of the proteins into the cell cytosol, and the receptor binding domain (RBD) (domain B) that encodes the combined repetitive oligopeptide (CROP) structures, which are thought to be required for the interaction of the toxins with host cell carbohydrate structures (10–12) to initiate toxin internalization.

A dual-receptor mechanism has been suggested for the LCTs (13). This mechanism involves the initial interaction of the LCT CROP domain with cell surface-associated oligosaccharides, followed by specific binding of the toxins to a second, high-affinity receptor (13). Early studies using *in vitro* assays indicated that TcdA bound to the trisaccharide Galα1-3Galβ1-4GlcNAc (10); however, this trisaccharide is not naturally found on human cells. Of more physiological relevance are the carbohydrate structures that include the Ii and Lewis X and Y antigens, which are present on human epithelial cells (14, 15) and which bind to the TcdA CROP region (16). As suggested by Schorch et al., all LCTs, including TcdB, are likely to use a similar binding mechanism to initiate host cell contact (13).

In addition to receptors for TcdA, which include human colonocyte membrane protein glycoprotein 96 (GP96) (17), several potential receptors for TcdB have also been identified (18–20). Poliovirus receptor-like 3 was shown to be required for TcdB-mediated cytotoxicity of both Caco-2 and HeLa cells and may serve as a TcdB receptor (19); however, a second study did not support these observations (20). Chondroitin sulfate proteoglycan 4 (CSPG4) was also identified as a possible TcdB receptor in two separate studies; however, the binding locations within the toxin differed between the two studies (18, 20). Most recently, members of the frizzled family of receptors (FZDs), required for Wnt signaling, were also identified as potential TcdB receptors; however, these appear to bind to TcdB outside the CROP region (20, 21).

Currently, there is no available vaccine for CDIs, but the toxins TcdA and TcdB are known to be immunogenic, and immune responses against these toxins can protect against reinfection (22, 23). Subunits of TcdA and TcdB have been successfully assessed as possible vaccine candidates (22). In this study, we aimed to identify the direct interaction between oligosaccharides and TcdA/TcdB using the different domains of the two toxins that have previously been show to elicit protection from CDIs.

## RESULTS

### Glycan array analysis of TcdA and TcdB domains.

**FIG 1:**
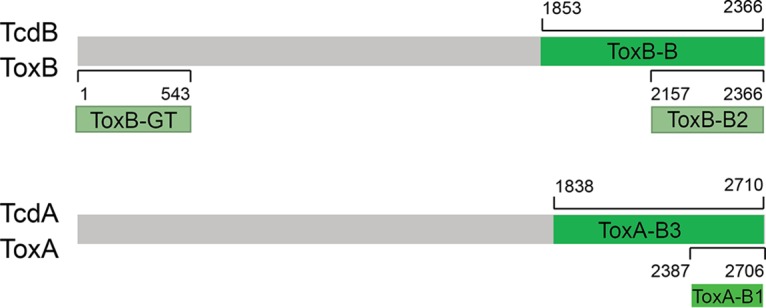
TcdA and TcdB domains and fragments used in this study. Numbers indicate amino acids of the full protein sequence.

TcdA and TcdB domains were analyzed using glycan arrays. The TcdA protein fragments tested were ToxA-B3 (binding domain) and ToxA-B1 (the last 209 amino acids of the binding domain). The TcdB fragments tested were ToxB-GT (first 181 amino acids of the GTPase domain) and ToxB-B2 (final 70 amino acids of the binding domain) (Fig. 1; see also Fig. S1 in the supplemental material).

The glycan array analysis revealed that ToxA-B1 and ToxA-B3 had overlapping binding on 20 glycan structures, while ToxA-B3 had additional binding to α/β-linked galactose and terminal *N*-acetylgalactosamine structures (Tables 1 and S1).

**TABLE 1:**
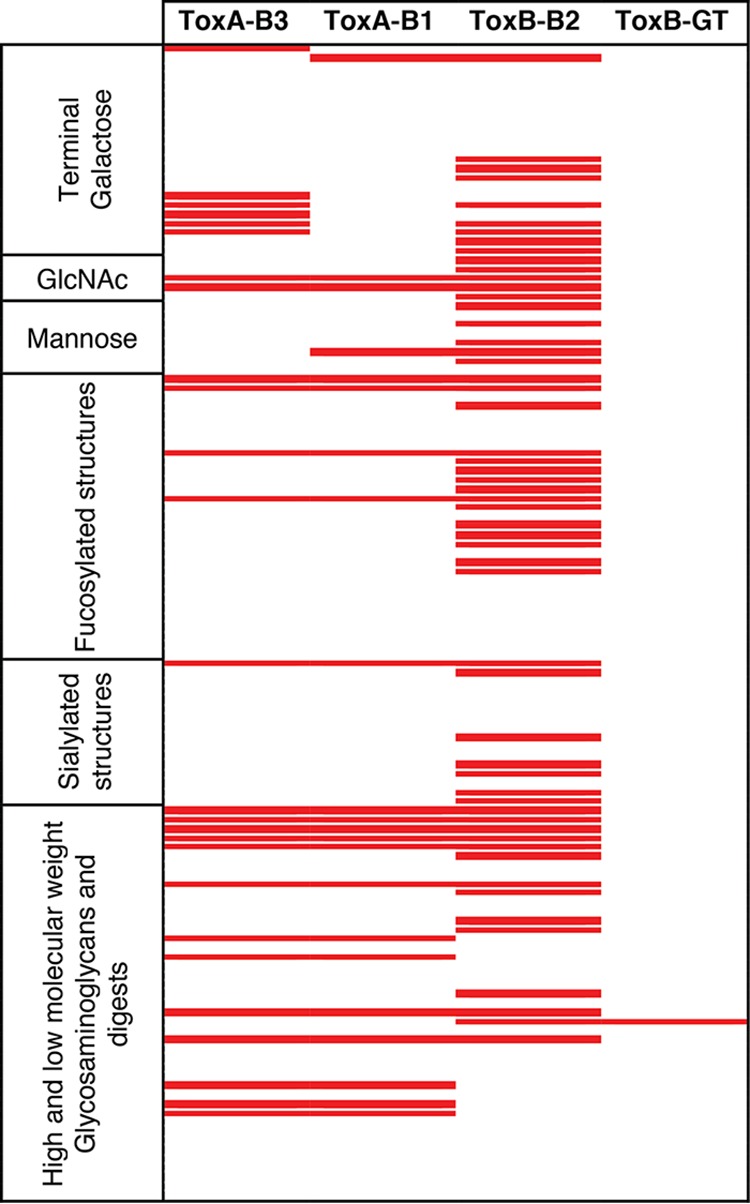
Glycan array analysis of TcdA and TcdB protein domain fragments^*a*^

a Red indicates binding. Binding was determined by positive interactions in three replicate array experiments. Positive interactions are defined as a fluorescence value significantly above the background fluorescence level (average background fluorescence from 20 spots +3 standard deviations). For full glycan names and structures, see Table S1 in the supplemental material.

The TcdB protein fragment ToxB-B2 had a much wider glycan structure recognition than TcdA fragments, binding to more than 50 structures of the 126 glycans printed on the array whereas the TcdA protein fragments recognized a maximum of 26 glycans (Tables 1 and S1). Binding for the TcdB proteins was spread across all the different terminal and side-branched oligosaccharides, showing no preference for a particular sugar moiety. The ToxB-GT fragment recognized only a single structure on the array (13H; GlcAβ1-3GlcNAcβ1-4n [*n* = 10]) (Tables 1 and S1).

### SPR of TcdA/B and glycans identified by array analysis.

To validate the glycan array results and to determine the dissociation equilibrium constant (*K_D_*) of the interactions, surface plasmon resonance (SPR) was performed between free oligosaccharides (blood group and Lewis antigens) and TcdA and TcdB domain proteins (Table 2). Both TcdA and TcdB binding domains had high-affinity interactions with blood group A oligosaccharide (ToxB-B2, 29.1 nM; ToxA-B3, 61.4 nM), while much poorer binding was observed for both proteins interacting with blood group B oligosaccharide (ToxB-B2, 42.9 μM; ToxA-B3, 14.4 μM) (Table 2). ToxB-B2 bound to nonsialylated Lewis A and X significantly better than to the equivalent sialyl-Lewis antigens (*P* < 0.02). The TcdA fragments ToxA-B1 and ToxA-B3 differentially recognized blood group H glycans on the array, with only tetrasaccharide H-glycan bound consistently by TcdA fragments (Table 1 and Fig. S3). Only the shorter ToxA-B1 recognized the blood group H trisaccharide used in the SPR, with a *K_D_* of 1.22 μM.

**TABLE 2:** SPR analysis of TcdA and TcdB toxin fragments

Antigen or oligosaccharide	Protein binding (*K_D_*)^*a*^		
	ToxA-B1	ToxA-B3	ToxB-B2
Blood group A	1.53 μM ± 0.6 μM	61.4 nM ± 29.6 nM	29.1 nM ± 7.5 nM
Blood group B	11.4 μM ± 1.08 μM	14.4 μM ± 3.2 μM	42.9 μM ± 59.1 μM
Blood group H	1.22 μM ± 0.49 μM	NCDI	428 nM ± 8 nM
Lewis A	3.38 μM ± 0.95 μM	1.65 μM ± 0.62 μM	579 nM ± 37 nM
Lewis B	1.05 μM ± 0.63 μM	8.01 μM ± 0.58 μM	395 nM ± 29 nM
Lewis Y	12.7 μM ± 2.47 μM	55.5 μM ± 28.8 μM	8 μM ± 0.74 μM
Lewis X	18.9 μM ± 5.58 μM	16.5 μM ± 2.8 μM	501 nM ± 308 nM
Sialyl-Lewis A	71.8 μM ± 27.1 μM	72.1 μM ± 31.8 μM	2.39 μM ± 0.68 μM
Sialyl-Lewis X	91.1 μM ± 4.16 μM	1.07 μM ± 0.15 μM	34.7 μM ± 11.5 μM
2-6SLN	NCDI	NCDI	105 nM ± 8.45 nM
α-Methyl-mannose	NCDI	NCDI	315 nM ± 103 nM
Man5^*b*^	NCDI	NCDI	NCDI

a *K_D_* is reported ±1 standard deviation. ToxB-GT was also tested with no concentration-dependent interaction observed for all glycans. NCDI, no concentration-dependent interaction observed up to the maximum concentration tested (100 μM). For representative curves, see Fig. S2 in the supplemental material.

b Mannopentaose.

### Lectin array analysis of Vero cells.

The Vero cells were examined for the presence of TcdB target glycans using lectin array analysis (Table S2). Lectins recognizing terminal β-linked galactose were observed as well as lectins recognizing GalNAc, Neu5Acα2-3/6Gal, mannose, and α-fucosylated structures. These data indicate that Vero cells express all of the structures recognized by TcdB in glycan array analysis.

### Inhibition of TcdB using free-oligosaccharide inhibitors.

**FIG 2:**
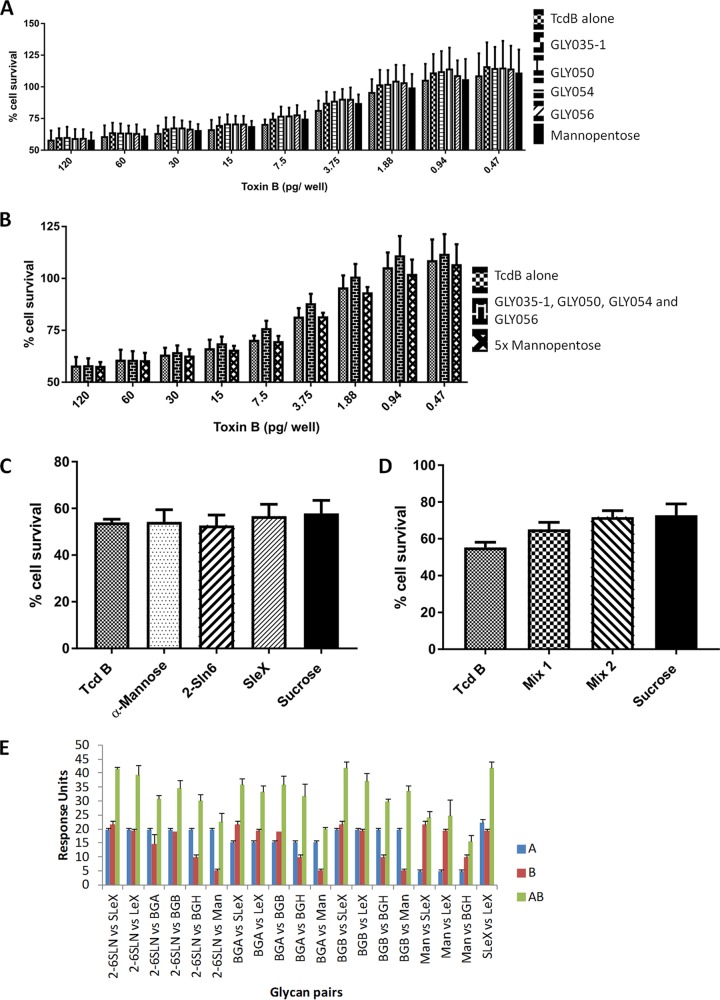
Cell viability MTS assays were used as a quantitative measure of cytotoxicity. Cell survival was calculated as the percent absorbance at 490 nm of each treated sample relative to that of the untreated sample. (A) Percent survival of Vero cells treated with TcdB alone versus that with TcdB containing each individual glycan: TcdB/GLY035-1, TcdB/GLY050, TcdB/GLY054, TcdB/GLY056, and TcdB/mannopentaose. (B) The percent survival of Vero cells treated with TcdB alone versus the mixed glycans (GLY035-1, GLY050, GLY054, and GLY056) and versus TcdB/mannopentaose (5× concentration). (C) Individual glycans based on the lectin analysis used at a single toxin concentration (125 pg/ml). (D) The data represent the average of three biological replicates, each performed in duplicate, with the error bars representing the standard errors of the means. ANOVA (Kruskal-Wallis) was performed on the data set, with individual differences detected using Bonferroni’s multiple comparisons; differences between results for TcdB alone or with negative-control glycan and those with TcdB with all glycans showed no statistical significance. (E) Summary of the SPR competition assay of the full-length TcdB protein showing the individual response units of each glycan when run first (blue/A) or second (red/B) or when combined (green/AB). All heterogeneous glycan pairs result in roughly the additive response of the two individual glycans, indicating no competition between glycans. BGA, blood group A glycan; BGB, blood group B glycan; BGH, blood group H/O glycan.

To determine if free oligosaccharides could reduce cytotoxicity of TcdB, Vero cell cytotoxicity assays were conducted, and cell viability was determined using a quantitative MTS [3,4-(5-dimethylthiazol-2-yl)-5-(3-carboxymethoxyphenyl)-2-(4-sulfophenyl)-2H-tetrazolium salt] assay. Blood group A type 1 (GLY035-1; array identifier [ID] 7K), blood group H (GLY030; array ID 7F), blood group B (GLY38-3; array ID 7M), Lewis X (GLY050; array ID 7I), Lewis A (GLY054; array ID 7J), Lewis B (GLY056; array ID 7P), sialyl-Lewis X (SLeX) (GLY053; array ID 10B), and α2-6 sialyllactosamine (2-6SLN) (SLN306; array ID 10L) were used in the assays individually and in combination, with mannopentaose (array ID 5H) used as a negative control. Here, we showed that preincubation of either blood group A type 1, Lewis X, Lewis A, and Lewis B with TcdB did not individually alter the cytotoxic potential of this toxin as Vero cell survival was comparable to that with the TcdB-alone control or the mannopentaose-TcdB combination (Fig. 2A and C). However, even when glycans were mixed with TcdB in different combinations, no significant blocking of toxin-mediated cell killing was observed (Fig. 2B and D).

As no glycans alone or in combination were able to block TcdB toxicity, a competition assay of the glycans was performed using a ForteBio Pioneer SPR system. NextStep single-injection competition analysis showed that preinjection of glycans, including Lewis X, SLeX, 2-6SLN, blood group antigens (A, B, or H), and mono-mannose, could not inhibit TcdB binding to a second heterogeneous glycan, indicating that TcdB has more than one glycan binding site (Fig. 2E and S2).

## DISCUSSION

Host glycosylation is a common target for bacterial pathogens (24–28). Of the bacterial virulence factors that recognize glycans, toxins from a wide range of bacterial pathogens have been identified that use host glycoconjugates to initiate contact and cytotoxicity (26, 27). TcdA has previously been reported to recognize the nonhuman glycoconjugate Galα1-3Galβ1-4GlcNAc (10). Previous screening of the TcdA/B domain by the Consortium for Functional Glycomics (CFG; http://www.functionalglycomics.org/glycomics/HServlet?operation=view&sideMenu=no&psId=primscreen_2693) and analysis of the full-length TcdA by electrospray mass spectrometry and molecular modeling (11, 12, 29) indicated that TcdA could also bind β-galactosidase (β-Gal)-containing structures with binding to Lewis A and Galβ1-4GlcNAc core structures, particularly those found in the most abundant human milk oligosaccharides (29). Our TcdA binding studies using the ToxA-B3 fragment showed remarkably similar binding with structures recognized by the fragment of TcdA used by the CFG, including Lewis antigens, blood group antigens, and terminal α- and β-galactose structures (Table 1). We also report the recognition of glycosaminoglycans and terminal *N*-acetylglucosamine glycoconjugates not observed by the CFG glycan array studies. This indicates that the full glycan binding profile of TcdA can be attributed to the binding domain as previously indicated by molecular modeling (11, 12, 29).

For TcdB, previous screening of the CFG glycan array was carried out on the full-length TcdB protein (http://www.functionalglycomics.org/glycomics/HServlet?operation=view&sideMenu=no&psId=primscreen_2694) and two C-terminal binding domain fragments, one equivalent to ToxB-B2 and a shorter C-terminal truncation of that fragment (http://www.functionalglycomics.org/glycomics/HServlet?operation=view&sideMenu=no&psId=primscreen_1593 and http://www.functionalglycomics.org/glycomics/HServlet?operation=view&sideMenu=no&psId=primscreen_1592). The TcdB analysis on the CFG array revealed binding only to nonhuman terminal α1-3Gal structures similar to those observed for TcdA but with much lower fluorescence values. These results are very different from the ones obtained on the glycan array reported here. The differences may be explained by the different surface chemistries used by the CFG (Schott Nexterion *N*-hydroxysuccinimide [NHS] three-dimensional [3D] polymer substrates) and the glycan array used in this study (ArrayIt SuperEpoxy II two-dimensional [2D] activated silicon surface). The isoelectric points of TcdA and its fragments (pI 8.7 to 8.9) and of TcdB and its fragments (pI 3.99 to 4.21) are vastly different. As the assay buffer used by the CFG is a standard pH 7.4 buffer, TcdA will have the opposite charge from TcdB in the assay buffer used. This indicates that TcdB may be incorrectly charged in the assay buffer to enter the 3D matrix present on the Schott Nexterion NHS slides favored by the CFG.

Molecular modeling of TcdB and mass spectroscopy using human milk oligosaccharides resulted in a glycan binding repertoire similar to that observed for TcdA (29). The results obtained for the binding domain of TcdB through glycan array analysis indicates that TcdB has a broader specificity than that observed for TcdA, with TcdB binding over 20 more glycans than TcdA. The additional glycans found to bind to TcdB included those that were sialylated and mannobiose glycans, providing a wider range of targets found in the host gastrointestinal tract for this toxin than for TcdA. The finding that TcdB has a more diverse glycan binding recognition than TcdA also correlates with the broader tissue tropism of TcdB demonstrated by Lanis et al. (30).

We observed a limited reduction of TcdB cytotoxicity using specific glycan structures in blocking assays. This result is very similar to that previously reported for human milk oligosaccharides (29). TcdB binds to a broad range of human glycans, allowing for engagement of a broad range of host cells (30). There are also multiple known protein receptors for TcdB. All of the protein targets, NECTIN3 (19), CSPG4 (18, 20), and FZD1/2/7 (20, 21), are glycoproteins that express a range of different glycosylations, including glycosaminoglycans (chondroitin) (18, 20) and complex N-linked glycans (31, 32). A recent study by Chen et al. demonstrated the structure of a region of TcdB (TcdB-FZD binding domain [FBD]) responsible for the interaction with FZD proteins outside the tested region of ToxB-B2 (21). The role of the N-linked glycans in the TcdB-FBD interaction have not been examined in this study as the region of FBD bound by TcdB did not express a typical N-linked glycan (21). However, the finding of an N-glycosylation site in proximity to the TcdB-FBD does give a possibility of an additional lectin site on the protein outside the ToxB-B region. A lectin site outside the ToxB-B2 fragment is of interest as the ToxB-B2 fragment appeared to have a bias toward glycosaminoglycans and glycans typically found as O-linked on proteins or on glycolipids or very short N-linked glycans rather than for components or cores of larger N-linked glycans. The facts that none of the glycoprotein binding has been linked to the ToxB-B2 region and that from the competition assay there are clearly multiple lectin sites on TcdB are consistent with the findings that free glycans could not significantly inhibit toxin activity at biologically acceptable levels. It is difficult to completely inhibit toxin function with a free glycan, even with combinations of glycans, when a large number of cellular targets are present, including all three of the known protein receptors (33). This phenomenon has been previously reported for pneumolysin as it was shown that inhibition of red blood cell (RBC) and epithelial cell cytotoxicity was possible using free sialyl-Lewis X (low-abundance target on RBCs and epithelial cells) but that the cytotoxicity of neutrophils that express an abundance of sialyl-Lewis X could not be inhibited (27).

The toxins from C. difficile, TcdA and TcdB, have broad glycan binding specificity that is determined by the binding domain of both toxins. There have been multiple distinct target glycans, proteins, and cell types reported for the Tcd proteins (4, 9–12, 16–20, 29). The broad specificity of the glycan binding of these toxins indicates that a number of different target molecules on multiple different cell types are potential targets for these proteins; thus, finding a single inhibitor capable of abrogating the cell damage mediated by the C. difficile toxins is unlikely.

## MATERIALS AND METHODS

### Cloning, expression, and purification of Tcd proteins.

ToxA-B1, ToxA-B3, ToxB-GT, and ToxB-B2 proteins were expressed and purified as previously described (22). Full-length TcdB was purchased from Abcam (ab124001).

### Glycan array.

Glycans were prepared for printing as described by Day et al. (34). Glycan array slides were printed SuperEpoxy 3-activated substrates using the glycan library previously described by Arndt et al. (35) and Day et al. (25). Table S1 in the supplemental material gives a full list of the structures printed. The glycan arrays were performed and analyzed as previously described (27). Briefly, 2 μg of protein in phosphate-buffered saline (PBS) (137 mM NaCl, 2.7 mM KCl, 10 mM Na_2_HPO_4_, 2 mM KH_2_PO_4_, pH 7.4) containing 1 mM MgCl_2_ and 1 mM CaCl_2_ was precomplexed with a mouse anti-His tag antibody (Cell signaling) and two Alexa 488-labeled antibodies (rabbit anti-mouse and goat anti-rabbit) at a molar ratio of 1:1:0.5:0.25 to enable detection. Protein was incubated on the slide for 30 min and washed three times in PBS. Slides were scanned on a PerkinElmer ProScan four-laser scanner and analyzed using ScanArray Express and Microsoft Excel.

### Surface plasmon resonance analysis.

The interactions between the Tcd protein fragments and test glycans were analyzed using surface plasmon resonance as described by Shewell et al. (27), with the following modifications. Proteins were immobilized onto a CM5 chip via amine coupling at pH 3.0 to 4.5 with a flow rate of 10 μl/min for 420 s, and an ethanolamine blank flow cell was used as a control. Glycans were tested between 160 nM and 100 μM. All data are double-reference subtracted.

### Inhibition of cytotoxicity of TcdB with free oligosaccharides.

To determine if blood group antigen A type 1 (GLY035-1), B type 5 (GLY038-3), H disaccharide (GLY030), Lewis X tetraose (GLY050), sialyl-Lewis X pentaose (GLY053), Lewis A tetraose (GLY054), Lewis B pentaose (GLY056; Elicityl Oligotech), and 2-6 sialylactosamine (SLN306; Dextra Laboratories), either individually or in combination, could reduce the cytotoxic effect of C. difficile TcdB on Vero cells, the following cytotoxicity assays were conducted. Vero cells were grown in culture flasks containing minimum essential medium alpha (MEMα; Life Technologies) with 10% heat-inactivated fetal calf serum (HI FCS), 100 units/ml penicillin, and 100 μg/ml streptomycin and incubated at 37°C in 5% CO_2_. The cells were then seeded at 1.0 × 10^5^ cells/ml in 96-well microtiter plates in MEMα supplemented with 1% HI FCS and subsequently used in cytotoxicity inhibition assays. Purified TcdB (ab124001; Abcam) was serially diluted 2-fold from 4 ng/ml to 0.015 ng/ml (toxin titer T1 to T9) and each dilution was used in the assay. GLY035-1, GLY050, GLY054, GLY056, and mannopentaose were each resuspended in 1 ml of sterile MilliQ water to yield a stock concentration of 6 mM for GLY035-1 and GLY056, 7.2 mM for GLY050 and GLY054, and 6.8 mM for mannopentaose. Each glycan was then diluted further in PBS and used in the cytotoxicity assay at a final concentration of 300 nM for GLY035-1, 5 μM for GLY050, 6 μM for GLY054 and mannopentaose, and 130 μM for GLY056. A total of 72 μl of each TcdB dilution (ranging from 4 ng/ml to 0.015 ng/ml) was then incubated with either 12 μl of culture medium (MEMα) and 36 μl of PBS alone (positive toxin control), 12 μl of glycan (GLY035-1, GLY050, GLY054, or GLY056) with 36 μl of PBS, or 48 μl of a combination of all glycans (12 μl each) excluding mannopentaose (which was instead used as a negative control in the assay) at room temperature (RT) for 45 min. Prior to addition of the glycan-toxin mixtures, Vero cells were preincubated in the presence of either culture medium, each individual glycan (at the same concentration stated above), or a combination of all 4 glycans (12 μl each; again at the same concentration stated above), excluding mannopentaose, for 10 min at RT. Mannopentaose or sucrose was also included at an equal concentration to the total moles of glycan as controls for the wells receiving the combination glycans in this assay. The toxin alone or toxin-glycan mixtures (50 μl) were then added to the cells, and the trays were incubated for 24 h at 37°C in 5% CO_2_. For α-mannose, 2-6SLN, SLeX, mix 1, and mix 2, 5 μl of each glycan (at a final concentration of 10 mM for individual glycans and 10 mM for each glycan in mix 1 [α-mannose and 2-6SLN] and mix 2 [2-6SLN and SLeX]) was added to 45 μl of culture medium. Sucrose at 10 mM was used as a negative control for these final glycans, and TcdB was used at a final concentration of 0.125 ng/ml. The Vero cells were then treated as described above and incubated as stated before.

### Cell viability assays.

To quantify cell viability in the tissue culture plates described above, a CellTiter 96-cell Proliferation Assay (Promega) was used, as previously described (36). Here, a 2-mg/ml solution of the tetrazolium compound 3-(4,5-dimethylthiazol-2-yl)-5-(3-carboxymethopheny)-2-(4-sulfophenyl)-2H-tetrazolium inner salt (MTS) (Sigma) was prepared in Dulbecco’s phosphate-buffered saline (DPBS) (2.7 mM KCl, 1.5 mM KH_2_PO_4_, 136.9 mM NaCl, 8.9 mM Na_2_HPO_4_ · 7H_2_O). A 0.92-mg/ml solution of the electron-coupling reagent phenazine methosulfate (PMS) (Sigma) was also prepared in DPBS. Prior to use, 100 μl of PMS was added to every 2 ml of MTS, and 20 μl of this mixture was added to each well of the assay. The plates were then incubated at 37°C in a 5% CO_2_ incubator and read at 490 nm after 1 h, using a Tecan infinite M200 plate reader. Cell viability or activity determined at this wavelength is directly proportional to the number of living (metabolically active) cells. The data represent the average of three biological replicates, each carried out in duplicate, and show the percentage of cell survival against the TcdB amount used (expressed as picograms of TcdB/well). Statistical analysis was performed using analysis of variance (ANOVA) (Kruskal-Wallis), with individual differences detected using Bonferroni’s multiple comparisons.

### Lectin array analysis of Vero cells.

The cells used for cytotoxicity assay, Vero cells, were analyzed for cell surface glycans using lectin arrays. Lectin arrays were printed using an ArrayJet Argus Marathon Inkjet Bio-Printing System on Arrayit SME3 substrates as previously described (37). Arrays were neutralized and performed as previously described (35, 37). Slides were scanned on an Innopsys InnoScan 1100AL to acquire the data of which lectins bound to the cells and analyzed using Innopsys Mapix data acquisition and analysis software and Microsoft Excel for statistical analysis (Student’s unpaired *t* test of fluorescence of background spots versus fluorescence of lectin-printed spots).

### Competition SPR.

Competition between different glycans for the binding of TcdB was performed using a ForteBio Pioneer SPR system. TcdB was loaded onto flow cell 1 of a COOH5 chip, and flow cell 2 was blank, immobilized to enable reference subtraction. OneStep and NextStep analyses of each of the glycans were programmed using the Pioneer instrument software package. Glycans were used at a concentration of 50 μM for both OneStep and NextStep analyses. OneStep was performed with a 70% loop volume and a 4% sucrose control. NextStep analysis was performed with a 45-s injection time with each glycan and PBS as the A component and with each glycan and PBS as the B component. Analyses of each cycle, OneStep and NextStep, were completed separately with a Qdat analysis software package.

## Supplementary Material

Supplemental file 1

Supplemental file 2

Supplemental file 3

Supplemental file 4

Supplemental file 5
