# Supplementary material for: Lectin Activity of the TcdA and TcdB Toxins of Clostridium difficile
Source: Infect Immun. 2019 Feb 21;87(3):e00676-18. doi: 10.1128/IAI.00676-18 (PMC6386544; doi:10.1128/IAI.00676-18)
Supplement: Supplemental file 1 [file IAI.00676-18-s0001.pdf]

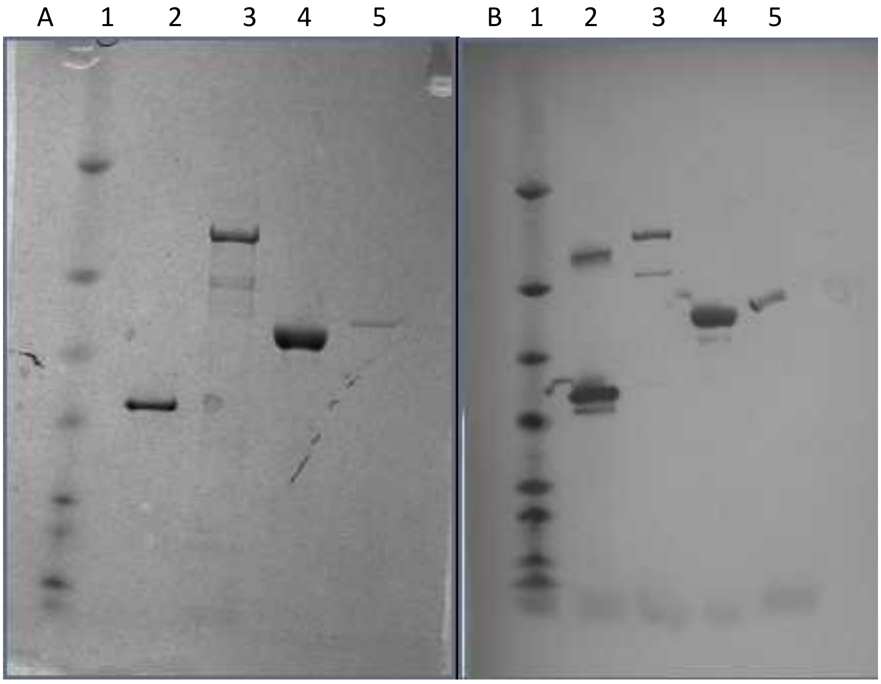

Figure S1 SDS-PAGE (A) and Western blot (B) of TcdA and TcdB protein fragments. Lane 1: Prestained ladder; Lane 2: ToxA (35kDA); Lane 3: ToxA-B3; Lane 4: ToxB-B2 (61kDA); Lane 5: ToxB2-GT.
