# Supplementary material for: Lectin Activity of the TcdA and TcdB Toxins of Clostridium difficile
Source: Infect Immun. 2019 Feb 21;87(3):e00676-18. doi: 10.1128/IAI.00676-18 (PMC6386544; doi:10.1128/IAI.00676-18)
Supplement: Supplemental file 2 [file IAI.00676-18-s0002.pdf]

TABLE S1. Red is binding white is no binding. Numbers indicate fold above background.

	Code	Name	Structure	ToxA-B3	ToxA-B1	ToxB-B2	ToxB-GT
Terminal Galactose	1A	Lacto-N-Biose I	Galβ1-3GlcNAc	1.6			
	1B	N-Acetyllactosamine	Galβ1-4GlcNAc	3.6	2.4	1.1	
	1C	β-1-4-galactosyl-galactose	Galβ1-4Gal				
	1D	β-1-6 Galactosyl-N-acetyl glucosamine	Galβ1-6GlcNAc				
	1E	β-1-3 Galactosyl-N-acetyl galactosamine	Galβ1-3GalNAc				
	1F	β-1-3 Gal-N-Acetyl galactosaminyl-β1-4 Gal-β1-4-Glc	Galβ1-3GalNAcβ1-4Galβ1-4Glc				
	1G	Lacto-N-tetraose	Galβ1-3GlcNAcβ1-3Galβ1-4Glc				
	1H	Lacto-N-neotetraose	Galβ1-4GlcNAcβ1-3Galβ1-4Glc				
	1I	Lacto-N-neohexaose	Galβ1-4GlcNAcβ1-6(Galβ1-4GlcNAcβ1-3)Galβ1-4Glc				
	1J	Lacto-N-hexaose	Galβ1-4GlcNAcβ1-6(Galβ1-3GlcNAcβ1-3)Galβ1-4Glc				
	1K	Globotriose	Galα1-4Galβ1-4Glc				
	1L	Tn Antigen	GalNAcα1-O-Ser				
	1M	Galactosyl-Tn Antigen	Galβ1-3GalNAcα1-O-Ser			1.6	
	1N	α1-3 Galactobiose	Galα1-3Gal			1.0	
	1O	Linear B-2 Trisaccharide	Galα1-3Galβ1-4GlcNAc			1.4	
	1P	Linear B-6 Trisaccharide	Galα1-3Galβ1-4Glc				
	2A	α1-3, β1-4,α1-3 Galactotetraose	Galα1-3Galβ1-4Galα1-3Gal	1.0			
	2B	Galβ1-6Gal	Galβ1-6Gal			1.0	
	2C	GalNAcβ1-3Gal	GalNAcβ1-3Gal	1.4			
	2D	GalNAcβ1-4Gal	GalNAcβ1-4Gal	1.0		1.6	
	2E	Galα1-4Galβ1-4GlcNAc	Galα1-4Galβ1-4GlcNAc	1.0		2.2	
	2F	GalNAcα1-3Galβ1-4Glc	GalNAcα1-3Galβ1-4Glc			2.6	
	2G	para-Lacto-N-hexaose (pLNH)	Galβ1-3GlcNAcβ1-3Galβ1-4GlcNAcβ1-3Galβ1-4Glc			1.3	
mine	4A	Diacetyl chitobiose	GlcNAcβ1-4GlcNAc			2.6	
	4B	Triacetyl chitotriose	GlcNAcβ1-4GlcNAcβ1-4GlcNAc			1.4	

Terminal N`Acetylglucosa	4C	Tetraacetyl chitotetraose	GlcNAcβ1-4GlcNAcβ1-4GlcNAcβ1-4GlcNAc	1.0	1.1	1.0
	4D	Hexaacetyl chitohexaose	GlcNAcβ1-4GlcNAcβ1-4GlcNAcβ1-4GlcNAcβ1-4GlcNAcβ1-4GlcNAc	1.0	2.7	2.9
	4E	Bacterial cell wall muramyl discaccharide	GlcNAcβ1-4MurNAc			1.4
Mannosyl	5A	β1-2 N-Acetylglucosamine-mannose	GlcNAcβ1-2Man			1.4
	5B	Bianntennary N-linked core pentasaccharide	GlcNAcβ1-2Manα1-6(GlcNAcβ1-2Manα1-3)Man			
	5C	α1-2-Mannobiose	Manα1-2Man			1.5
	5D	α1-3-Mannobiose	Manα1-3Man			
	5E	α1-4-Mannobiose	Manα1-4Man			1.3
	5F	α1-6-Mannobiose	Manα1-6Man		7.2	1.1
	5G	α1-3,α1-6-Mannotriose	Manα1-6(Manα1-3)Man			1.0
	5H	α1-3,α1-3,α1-6-Mannopentaose	Manα1-6(Manα1-3)Manα1-6(Manα1-3)Man			
	7A	Lacto-N-fucopentaose I	Fucα1-2Galβ1-3GlcNAcβ1-3Galβ1-4Glc	1.9	2.0	1.0
	7B	Lacto-N-fucopentaose II	Galβ1-3(Fucα1-4)GlcNAcβ1-3Galβ1-4Glc	1.6	2.2	2.4
	7C	Lacto-N-fucopentaose III	Galβ1-4(Fucα1-3)GlcNAcβ1-3Galβ1-4Glc			
	7D	Lacto-N-difucohexaose I	Fucα1-2Galβ1-3(Fucα1-4)GlcNAcβ1-3Galβ1-4Glc			1.8
	7E	Lacto-N-difucohexaose II	Galβ1-3(Fucα1-4)GlcNAcβ1-3Galβ1-4(Fucα1-3)Glc			
	7F	H-disaccaride	Fucα1-2Gal			
	7G	2'-Fucosyllactose	Fucα1-2Galβ1-4Glc			
	7H	3'-Fucosyllactose	Galβ1-4(Fucα1-3)Glc			
	7I	Lewisx	Galβ1-4(Fucα1-3)GlcNAc	2.1	2.3	3.7
	7J	Lewisa	Galβ1-3(Fucα1-4)GlcNAc			
	7K	Blood Group A trisaccharide	GalNAcα1-3(Fucα1-2)Gal			1.4
	7L	Lactodifucotetraose (LDFT)	Fucα1-2Galβ1-4(Fucα1-3)Glc			2.0

Fucosylated structures	7M	Blood Group B Trisaccharide	Galβ1-3(Fucα1-2)Gal		2.3
	7N	Lewisy	Fucα1-2Galβ1-4(Fucα1-3)GlcNAc	1.9	3.0
	7O	Blood Group H Type II Trisaccharide	Fucα1-2Galβ1-3GlcNAc		2.2
	7P	Lewisb tetrasaccharide	Fucα1-2Galβ1-3(Fucα1-4)GlcNAc		
	8A	Sulpho Lewisa	SO_3-3Galβ1-3(Fucα1-4)GlcNAc		1.3
	8B	Sulpho Lewisx	SO_3-3Galβ1-4(Fucα1-3)GlcNAc		1.7
	8C	Monofucosyl-para-Lacto-N-hexaose IV	Galβ1-3GlcNAcβ1-3Galβ1-4(Fucα1-3)GlcNAcβ1-3Galβ1-4Glc		1.8
	8D	Monofucosyllacto-N-hexaose III	Galβ1-4(Fucα1-3)GlcNAcβ1-6(Galβ1-3GlcNAcβ1-3)Galβ1-4Glc		
	8E	Difucosyllacto-N-hexaose	Galβ1-4(Fucα1-3)GlcNAcβ1-6(Fucα1-2Galβ1-3GlcNAcβ1-3)Galβ1-4Glc		1.5
	8F	Trifucosyllacto-N-hexaose	Galβ1-4(Fucα1-3)GlcNAcβ1-6(Fucα1-2Galβ1-3(Fucα1-4)GlcNAcβ1-3)Galβ1-4Glc		1.0
	8G	difucosyl-para-lacto-Nhexasoe II	Galβ1-4GlcNAcβ1-3Galβ1-4(Fucα1-3)Glc		
	8H	Lacto-N-neodifucohexaose I (LNnDFH I)	Fucα1-2Galβ1-4(Fucα1-3)GlcNAcβ1-3Galβ1-4Glc		
	8I	Lacto-N-neodifucohexaose II (LNnDFH II)	Fucα1-3Galβ1-4GlcNAcβ1-3Galβ1-4(Fucα1-3)Glc		
	8J	Trifucosyllacto-N-neoteraose I (TFLNnTI)	Fuca1-2Galb1-4(Fuca1-3)GlcNAcb1-3(Fuca1-2)Galb1-4Glc		
	8K	Monofucosyllacto- N-neohexaose I (MFLNnH I)	Galβ1-4(Fucα1-3)GlcNAcβ1-6(Galβ1-4GlcNAcβ1-3)Galβ1-4Glc		
	8L	Difucosyllacto-N-neohexaose I (DFLNnH I)	Galb1-4(Fuca1-3)GlcNAcb1-6(Galb1-4(Fuca1-3)GlcNAcb1-3)Galb1-4Glc		
	8M	Difucosyllacto-N-neohexaose II (DFLNnH II)	Fuca1-2Galb1-4(Fuca1-3)GlcNAcb1-6(Galb1-4GlcNAcb1-3)Galb1-4Glc		

	8N	Monofucosyl(1-3)-iso-lacto-N-octaose (MFiLNO)	Galb1-3GlcNAcb1-3Galb1-4(Fuca1-3)GlcNAcb1-6(Galb1-3GlcNAcb1-3)Galb1-4Glc		
	8O	Trifucosyl(1-2,1-2,1-3)-iso-lacto-N-octaose (TFiLNO (1-2,1-2,1-3))	Fucα1-2Galβ1-3GlcNAcβ1-3Galβb1-4(Fucα1-3)GlcNAcβ1-6(Galβ1-3GlcNAcβ1-3)Galβ1-4Glc		
Sialylated structures	10A	Sialyl Lewis a (S Lea)	Neu5Acα2-3Galβ1-3(Fucα1-4)GlcNAc	1.9	2.9
	10B	Sialyl Lewis x (S Lex)	Neu5Acα2-3Galβ1-4(Fucα1-3)GlcNAc		3.0
	10C	Sialyllacto-N-tetraose a	Neu5Acα2-3Galβ1-3GlcNAcβ1-3Galβ1-4Glc		
	10D	Monosialyl, monofucosyllacto-N-neohexose	Galβ1-4(Fucα1-3)GlcNAcβ1-6(Neu5Acα2-6Galβ1-4GlcNAcβ1-3)Galβ1-4Glc		
	10E	Disialyl-TF	Neu5Acα2-3Galβ1-3(Neu5Acα2-6)GalNAc		
	10H	Sialyllacto-N-fucopentaose VI (SLNFPVI)	Neu5Acα2-6Galβ1-3GlcNAcβ1-3Galβ1-4(Fucα1-3)Glc		
	10I	Monosialyllacto-N-hexaose (MSLNH)	Galβ1-3GlcNAcβ1-3(Neu5Acα2-6Galβ1-4GlcNAcβ1-6)Galβ1-4Glc		
	10J	Monosialyllacto-N-neohexaose (MSLNnH)	Neu5Acα2-6Galβ1-3GlcNAcβ1-3(Galβ1-4GlcNAcβ1-6)Galβ1-4Glc		
	10K	3'-Sialyllactosamine	Neu5Acα2-3Galβ1-4GlcNAc		1.9
	10L	6'-Sialyllactosamine	Neu5Acα2-6Galβ1-4GlcNAc		
	10M	LS-Tetrasaccharide a (LSTa)	Neu5Acα2-3Galβ1-3GlcNAcβ1-3Galβ1-4Glc		
	10N	LS-Tetrasaccharide b (LSTb)	Galβ1-3(Neu5Acα2-6)GlcNAcβ1-3Galβ1-4Glc		1.1
	10O	LS-Tetrasaccharide c (LSTc)	Neu5Acα2-6Galβ1-4GlcNAcβ1-3Galβ1-4Glc		1.7
	10P	Disialyllacto-N-tetraose	Neu5Acα2-3Galβ1-3(Neu5Acα2-6)GlcNAcβ1-3Galβ1-4Glc		
	11A	3'-Sialyllactose	Neu5Acα2-3Galβ1-4Glc		1.3

	11B	6'-Sialyllactose	Neu5Acα2-6Galβ1-4Glc			1.8
	12A	Neocarratetraose-41, 3-di-O-sulphate (Na+)	$C_{24}H_{36}O\text{-}_{25}S_{2}Na_{2}$ (Mixed anomers. Tetrasaccharide of regular κ - carrageenan)	2.4	4.7	2.8
	12B	Neocarratetraose-41-O-sulphate (Na+)	$C_{24}H_{37}O_{22}SNa$ (Mixed anomers. Derived from C1003 by removal of the non-reducing terminal 4-sulphate)	2.2	2.6	1.3
	12C	Neocarrahexaose-24,41, 3, 5-tetra-O-sulphate (Na+)	$C_{36}H_{52}O_{40}S_{4}Na_{4}$ (Mixed anomers. A hybrid sequence comprising carrageenan disaccharides in the order k-i-k, derived from the carrageenan from Chondrus crispus)	1.4	4.1	1.7
	12D	Neocarrahexaose-41, 3, 5-tri-O-sulphate (Na+)	$C_{36}H_{53}O_{37}S_{3}Na_{3}$ (Mixed anomers. Hexasaccharide of regular κ-carrageenan)	1.0	3.0	1.1
	12E	Neocarraoctaose-41, 3, 5, 7-tetra-O-sulphate (Na+)	$C_{48}H_{70}O_{49}S_{4}Na_{4}$ (Mixed anomers. Octasaccharide of regular κ-carrageenan)	2.4	2.4	1.4
	12F	Neocarradecaose-41, 3,5, 7, 9-penta-O-sulphate (Na+)	$C_{60}H_{87}O_{61}S_{5}Na_{5}$ (Mixed anomers. Decasaccharide of regular κ- carrageenan)			
	12G	ΔUA-2S-GlcNS-6S	$C_{12}H_{15}NO_{19}S_{3}Na_{4}$ (Predominant disaccharide produced from heparin by heparinase I and II)			
	12H	ΔUA-GlucNS-6S	$C_{12}H_{16}NO_{16}S_{2}Na_{3}$ (Produced from heparinase II digestion of heparin and heparin sulphate)			
	12I	ΔUA-2S-GlucNS	$C_{12}H_{16}NO_{16}S_{2}Na_{3}$ (Produced from heparin by digestion with heparinase I and II)	1.6	2.2	1.3
	12J	ΔUA-2S-GlcNAc-6S	$C_{14}H_{18}NO_{17}S_{2}Na_{3}$ (Minor component produced from heparin by heparinase II)			1.8
	12K	ΔUA-GlcNAc-6S	$C_{14}H_{19}NO_{14}SNa_{2}$ (Product of the action of heparinases II and III on heparin and heparan sulphate)			

High and low molecular weight Glycosaminoglycans and digests	12L	ΔUA-2S-GlcNAc	$C_{14}H_{19}NO_{14}SNa_2$ (Minor product of the action of heparinase II on heparin)		
	12M	ΔUA-GlcNAc	$C_{14}H_{20}NO_{11}Na$ (Produced from heparin sulphate by digestion With heparinase III)		2.8
	12N	ΔUA-GalNAc-4S (Delta Di-4S)	$C_{14}H_{19}NO_{14}SNa_2$ (Produced from various chondroitin sulphates By the action of chondroitinases ABC, B and AC-1)		1.0
	12O	ΔUA-GalNAc-6S (Delta Di-6S)	$C_{14}H_{19}NO_{14}SNa_2$ (Produced from various chondroitin sulphates By the action of chondroitinases ABC, AC-1 and C)	1.3	
	12P	ΔUA-GalNAc-4S,6S (Delta Di-disE)	$C_{14}H_{18}NO_{17}S_2Na_3$ (Produced from various chondroitin sulphates By the action of chondroitinases ABC, B and AC-1)		
	13A	ΔUA-2S-GalNAc-4S (Delta Di-disB)	$C_{14}H_{18}NO_{17}S_2Na_3$ (Produced from various chondroitin sulphates by action of chondroitinase ABC and/or B. Most typically from chondroitin sulphate B (dermatan sulphate))	2.3	1.4
	13B	ΔUA-2S-GalNAc-6S (Delta Di-disD)	$C_{14}H_{18}NO_{17}S_2Na_3$ (Produced from various chondroitin sulphates by the action of chondroitinase ABC)		
	13C	ΔUA-2S-GalNAc-4S-6S (Delta Di-tisS)	$C_{14}H_{17}NO_{20}S_3Na_4$ (Produced as a minor component by the action of chondroitinase ABC on various chondroitin sulphates, particularly B)		
	13D	ΔUA-2S-GalNAc-6S (Delta Di-UA2S)	$C_{14}H_{19}NO_{14}SNa_2$ (Produced as a minor component from various chondroitin sulphates by the action of chondroitinase ABC)		
	13E	ΔUA-GlcNAc (Delta Di-HA)	$C_{14}H_{20}NO_{11}Na$ (The only unsaturated disaccharide produced from hyaluronic acid by the action of chondroitinase ABC or AC-1)		1.7
	13F	Hyaluronan fragment (4mer)	(GlcAβ1-3GlcNAcβ1-4)n (n=4)		

	13G	Hyaluronan fragment (8mer)	(GlcAβ1-3GlcNAcβ1-4)n (n=8)	1.2	1.6		
	13H	Hyaluronan fragment (10mer)	(GlcAβ1-3GlcNAcβ1-4)n (n=10)				3.4
	13I	Hyaluronan fragment (12mer)	(GlcAβ1-3GlcNAcβ1-4)n (n=12)				
	13J	Heparin	(GlcA/IdoAα/β1-4GlcNAcα1-4)n (n=200)	1.0	1.8		
	13K	Chondroitin sulfate	(GlcA/IdoAβ1-3(±4/6S)GalNAcβ1-4)n (n<250)				
	13L	Dermatan sulfate	((±2S)GlcA/IdoAα/b1-3(±4S)GalNAcβ1-4)n (n<250)				
	13M	Chondroitin 6-sulfate	(GlcA/IdoAβ1-3(±6S)GalNAcβ1-4)n (n<250)				
	13N	HA-4 10mM	(GlcAβ1-3GlcNAcβ1-4)n (n=4)				
	13O	HA-6 10mM	(GlcAβ1-3GlcNAcβ1-4)n (n=6)	1.1	2.5		
	13P	HA-8 9.7mM	(GlcAβ1-3GlcNAcβ1-4)n (n=8)				
	14A	HA-10 7.83mM	(GlcAβ1-3GlcNAcβ1-4)n (n=10)		1.4		
	14B	HA-12 6.5mM	(GlcAβ1-3GlcNAcβ1-4)n (n=12)		2.1		
	14C	HA-14 5.6mM	(GlcAβ1-3GlcNAcβ1-4)n (n=14)				
	14D	HA-16 4.9mM	(GlcAβ1-3GlcNAcβ1-4)n (n=16)				
	14E	HA 30000Da 2.5mg/mL	(GlcAβ1-3GlcNAcβ1-4)				
	14F	HA 107000Da 2.5mg/mL	(GlcAβ1-3GlcNAcβ1-4)				
	14G	HA 190000Da 2.5mg/mL	(GlcAβ1-3GlcNAcβ1-4)				
	14H	HA 222000Da 2.5mg/mL	(GlcAβ1-3GlcNAcβ1-4)				
	14I	HA 1600000Da 2.5mg/mL	(GlcAβ1-3GlcNAcβ1-4)				
	14J	heparin sulphate 5mg/ml					
	14K	beta1-3Glucan					
