# Supplementary figures and images for: Lectin Activity of the TcdA and TcdB Toxins of Clostridium difficile
Source: Infect Immun. 2019 Feb 21;87(3):e00676-18. doi: 10.1128/IAI.00676-18 (PMC6386544; doi:10.1128/IAI.00676-18)

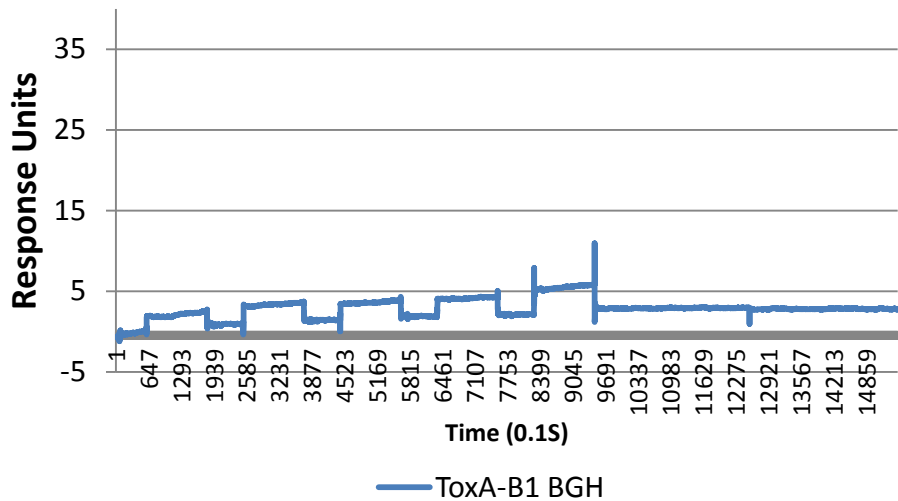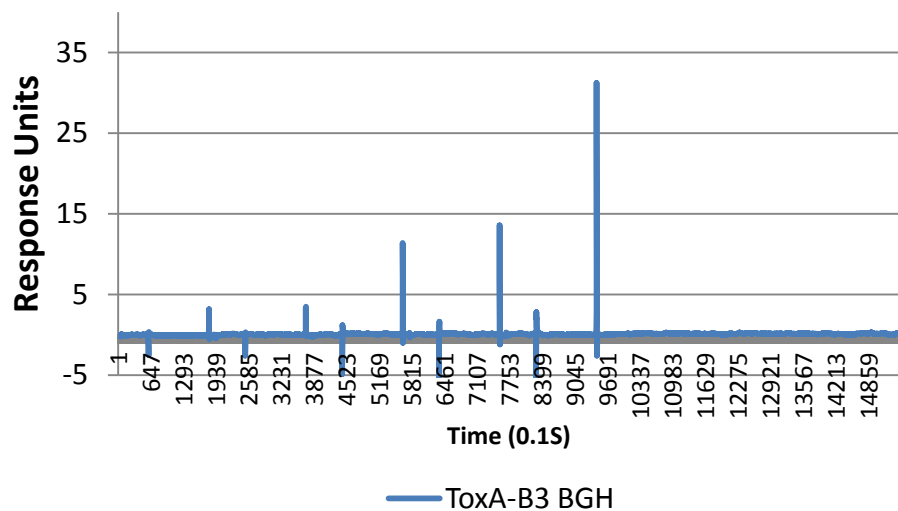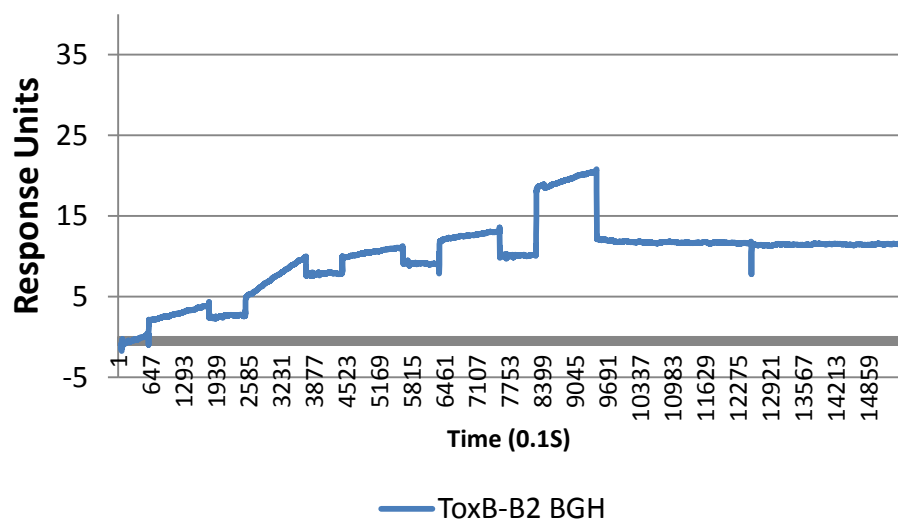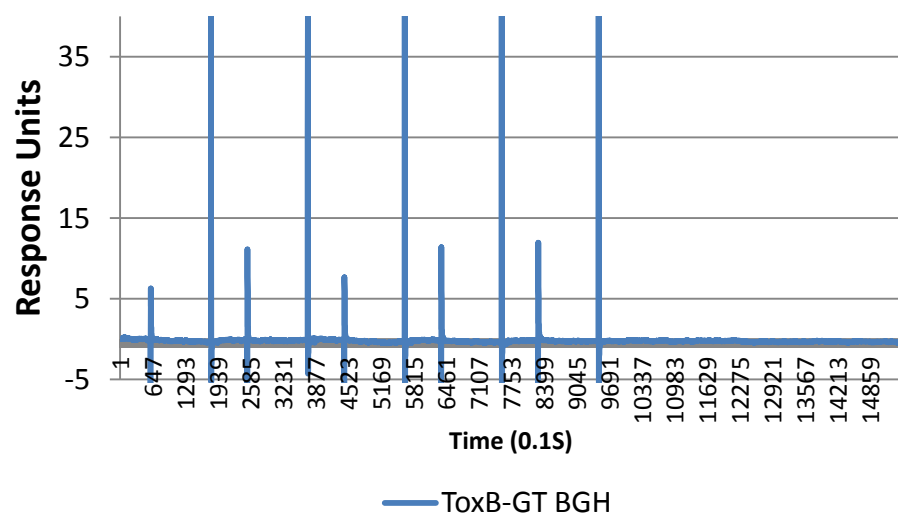

**Figure S2 Representative SPR curves for blood group H antigen for all four proteins tested.**

Supplement: Supplemental file 3 [file IAI.00676-18-s0003.pdf]

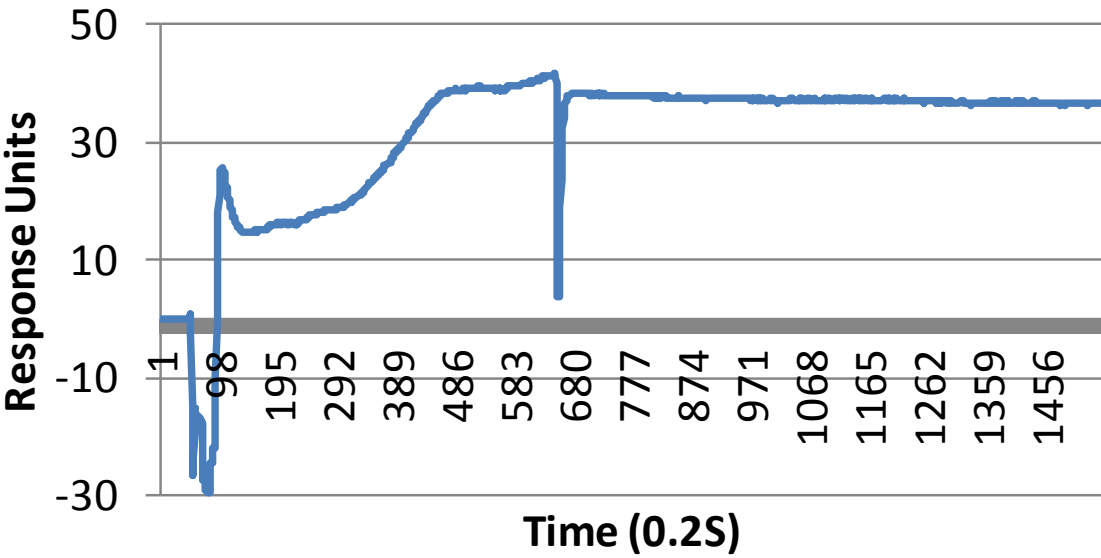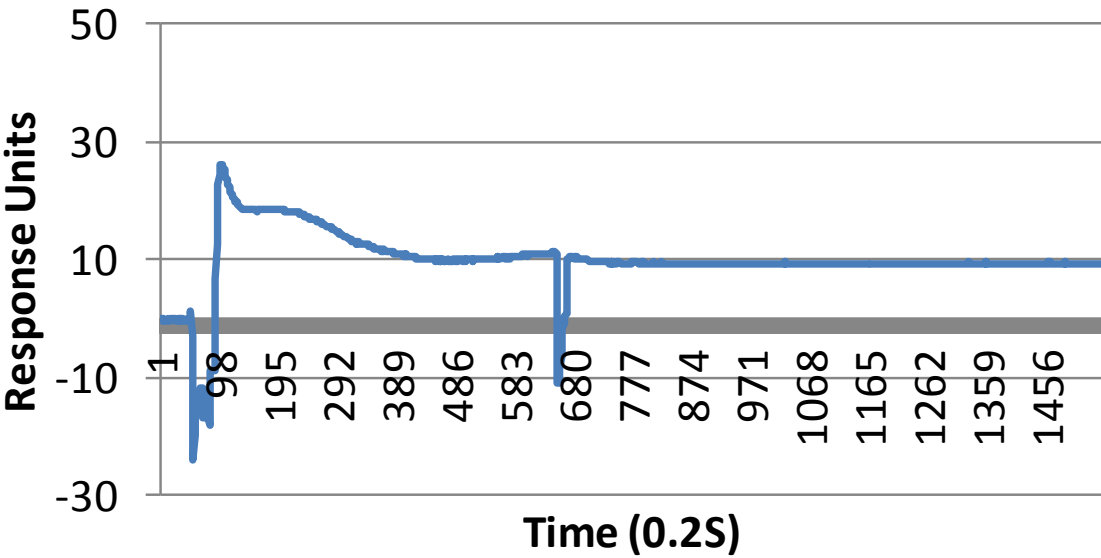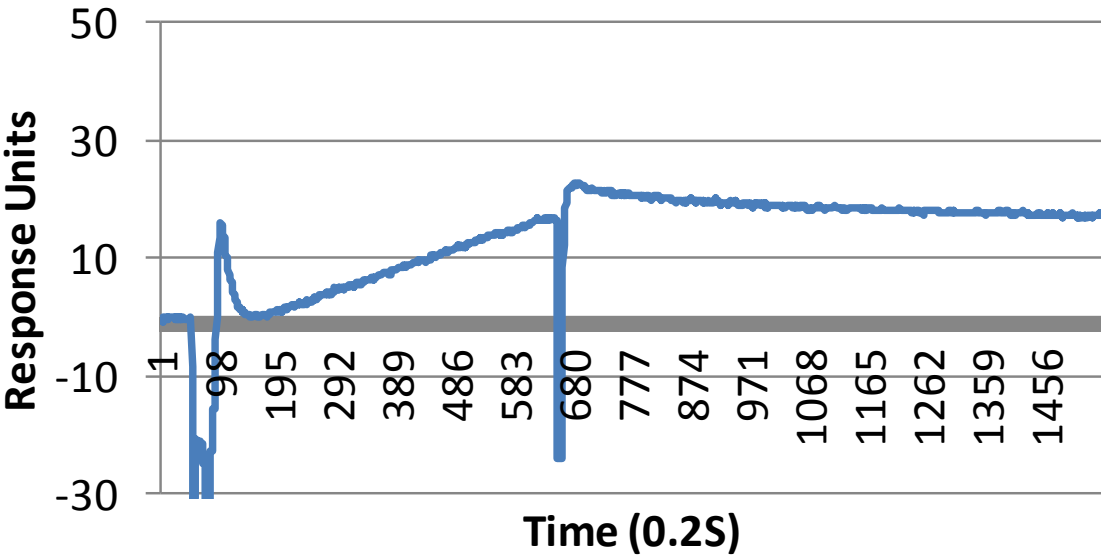

Figure S3 Representative SPR competition curves for 2-6SLN and SLeX.

Supplement: Supplemental file 5 [file IAI.00676-18-s0005.pdf]
