# Supplementary material for: Lectin Activity of the TcdA and TcdB Toxins of Clostridium difficile
Source: Infect Immun. 2019 Feb 21;87(3):e00676-18. doi: 10.1128/IAI.00676-18 (PMC6386544; doi:10.1128/IAI.00676-18)
Supplement: Supplemental file 4 [file IAI.00676-18-s0004.pdf]

**Lectin**	**Binds to**	Average	SEM
AAA	Fucα1-2[a]	6875.32	1143.24
ABA	Galβ1-3GalNAc[a]	364.42	872.30
ACA	Galβ1-3GalNAc[a]	289.00	282.99
AMA	Manα[a]	146.21	281.99
ASA	Manα1-3[a]	116.89	350.32
BC2LA	Manα[b]	65.93	235.31
BDA	GalNAcα, GalNAcβ[a]	2634.50	1300.50
BPA	GalNAc[a]	1031.94	330.71
BS-I	Galα, GalNAcα[a]	1276.94	458.50
CA	Galβ1-4GlcNAc, GalNAcβ1-4GlcNAc[a]	4219.03	1586.56
CAA	GlcNAcβ1-2Manα1-3(GlcNAcβ1-2Manα1-6)Manβ1-4GlcNAcβ1-4GlcNAcβ[a]	3805.50	1637.84
CALSEPA	Man, Glc, Glcα1-4Glc[a]	2542.84	742.91
CCA	Neu5Ac[a]	3310.16	722.88
CFA	GalNAc[c]	26.31	511.46
ConA	Man (Terminal, Branched), GlcNAc (Terminal)[a]	769.53	565.88
CPA	Manα Man[a]	2471.69	780.86
CSA	GalNAcα (Terminal)[a]	1821.78	722.72
DBA	GalNAcα1-3GalNAc, GalNAcα1-3Gal[a]	2186.19	854.47
ECA	Galβ1-4GlcNAc (Terminal)[a]	2162.31	556.06
EEA	GalNAcβ[a]	3297.72	975.36
GHA	Galα, GalNAcα[a]	33.97	173.44
GNA	Manα (Terminal)[a]	3204.78	561.68
GS-I-A4	GlcNAcα, GlcNAcβ[a]	142.16	452.00
GS-I-B4	GalNAcα[a]	259.78	946.51
GS-II	Galα (Terminal)[a]	254.22	473.30
HAA	GlcNAcα, GalNAcα[a]	3267.94	452.62
HHA	Manα (Terminal)[a]	408.38	541.51
HMA	GalNAcα, Fucα, Neu5Ac[a]	594.44	1047.58
HPA	GalNAcα (Terminal)[a]	316.41	880.45
IAA	GalNAc[a]	1819.75	888.49
IRA	GalNAcα, GalNAcβ[a]	2139.13	944.97
JACLIN	Galα, Galβ, GalNAcα (O-linkage)[a]	1235.44	430.97
LAA	GlcNAcβ, GlcNAcβ1-4GlcNAc[a]	222.09	415.24
LAL	Fuc[a]	96.72	477.47
LBA	GalNAcα, GalNAcα1-3(Fucα1-2)Gal[a]	2210.41	607.69
LcH	complex (Man/GlcNAc core with Fuca1-6)[a]	56.44	342.80
LcHB	α-Man> Glc, GlcNAc[a]	1963.94	532.25
LEA	GlcNAc-β(1,4)GlcNAc[a]	7814.50	1584.19
LFA	Neu5Ac[a]	328.84	728.92
LOTUS	Fuc[a]	3177.68	936.13
LPA	Neu5Ac[a]	3061.85	1314.00
MAA	Neu5Acα2-3Gal[a]	1677.38	235.38
MIA	Unknown[a]	971.00	2414.18
MNA-G	Galα, Galβ[a]	155.03	273.57

MNA-M	Manα[a]	1889.13	752.31
MOA	Galα1-3[a]	1836.38	469.94
MPA	Galα, GalNAcα[a]	1413.47	2938.29
NPA	Manα Manβ[a]	4458.05	579.28
PAA	Unknown[a]	448.66	854.66
PA-IIL	Lewis A > Fuc> Man[b]	1617.69	453.86
PA-IL	Galα, Galβ[b]	84.25	264.11
PEA	Manα, Glcα, GlcNAcα[a]	5217.28	1858.54
PHA-E	Galβ1-4GlcNAcβ1-2(Galβ1-4GlcNAcβ1-6)Man[a]	683.50	1478.19
PHA-L	Galβ1-4GlcNAcβ1-2Man[a]	4182.06	790.04
PHA-M	Unknown[a]	688.66	870.98
PHA-P	Unknown[a]	167.13	376.69
PMA	Manα1-3[a]	565.25	761.84
PNA	Galβ (Terminal)[a]	290.72	632.44
PSL	Neu5Acα2-6Galβ1-4GlcNAc, Neu5Acα2-6Galβ1-4Glc[a]	128.38	319.12
PTA-GAL	Gal[a]	3563.13	1029.09
PTA-GalN	GalNAc[a]	101.97	830.87
PWM	GlcNAcβ1-4GlcNAc[a]	371.09	540.08
RPA	complex[a]	3564.19	416.00
RSL	αFuc1-2Gal>αFuc1-6GlcNAC >αFuc1-4GlcNAC[b]	525.22	912.74
RTA	Glc[a]	156.13	820.08
SBA	GalNAcα (Terminal), Neu5Acα2-6GalNAc (Tn antigen)[a]	842.78	357.84
SHA	GalNAc[a]	3724.13	664.97
SJL	GalNAc(α1-6)Gal[a]	112.56	293.99
SNA-I	Neu5Acα2-6Gal, Neu5Acα2-6GalNAc[a]	2810.38	207.44
SNA-II	Galβ (Terminal), GalNAcβ (Terminal) [a]	3357.49	1223.69
SSA	GalNAc, Terminal, O-link[a]	83.72	192.37
STA	GalNAc, [GlcNAc-β(1,4)]$_2$[a]	161.91	282.62
Succ ConA	Manα, Glcα[a]	1156.34	285.22
Succ WGA	[GlcNAc-β(1,4)]$_2$[a]	3460.50	476.20
TKA	Galβ, Galβ1-4Glc (Lactose) [a]	417.97	725.03
TL	α-GalNAc,β-GalNAc, GalNAc, Gal, Fucose[a]	760.22	801.27
UDA	GalNAcβ[a]	497.72	318.85
UEA-I	Fucα[a]	168.63	464.07
UEA-II	GlcNAcβ, Fucα1-2Galβ1-4GlcNAc[a]	15.50	236.60
VAA	Galβ[a]	1875.19	183.81
VFA	Manα[a]	574.53	887.66
VGA	Galβ1-3GalNAc[a]	249.59	564.73
VRA	Galα [a]	1842.52	247.84
VVA	GalNAcα, GalNAcα1-3Gal[a]	2677.30	138.22
VVA MAN	Man[a]	638.09	597.43
WFA	GalNAcα, GalNAcβ[a]	510.06	846.15
WGA	GlcNAcβ[a]	363.25	396.97

Red indicates binding significantly above background (P<0.01),
white indicates no binding above background to the lectin on the array.
Arrays were repeated at least twice with four spots per lectin per array.

[abc] Binding specificities are as per suppliers data sheets.

[a]Obtained from EY Laboratories.

[b]Obtained from Elicityl.

[c]Obtained from Sigma-Aldrich.
